# Address

**Published:** 1886-07

**Authors:** Geo. J. Friedrichs


### Address.

BY GEO. J. FRIEDRICHS, M.D., D.D.S.

(Read before the Louisiana State Dental Society, March 4th, 1886.)

*Members of the Louisiana State Dental Society*:

Gentlemen—Called by your suffrages to preside over your deliberations and conformably to a provision of the Constitution of our Society, as well as the sanction of time-honored custom, which requires the incumbent of so distinguished an honor, to deliver an annual address, I know of no theme more appropriate, than the Louisiana State Dental Society—the part it must play in the regulation of the practice of dentistry and the influence it is destined to exert in the development and elevation of dental and oral science in the State of Louisiana.

The records of this organization show that it first saw the light of day on the 17th of June, 1878, when a few of us, practicing in the city of New Orleans, feeling the necessity of focalizing the light in the dental profession in our State and of elevating that profession to its proper dignity among the arts and sciences, banded ourselves together to accomplish and maintain these objects, so laudable and so desirable. You all know what were the trials and vicissitudes which have beset this Society in the past. How the grand old ship has been battered by the winds of adversity and all but wrecked upon the rocks and shoals of professional dissention and misunderstanding; how she has weathered every storm and has at last arrived in a port of safety; been docked upon the ways of fraternal good will and her timbers strengthened in every particular. New furnished and new manned throughout, she now spreads her sails, her banners unfurled to the breeze to venture forth in quest of new discoveries upon the sea of science.

Gentlemen, I regard this event as the proudest moment of my life, contemplating as I do a reunited profession; all asperities smoothened, the hand of fellowship mutually extended; binding us again under the ægis of one brotherhood, more firmly resolved than ever to advance the interests and well being of the science and art of dentistry in the State of Louisiana. We have realized how

> "Sweet are the uses of adversity;
> Which, like the toad, ugly and venomous,
> Wears yet a precious jewel in his head."

Long have I looked forward to this result, and now my hopes, have expanded into fruition. Our Society is just entering upon the great labor of its life. The hill top which seemed so difficult of access has at last been reached; and you are now enjoying the fragrance and beauty of its accustomed flowers. Before you in long perspective stretches the untried future. The haze veils the features of the landscape, but the haze only adds to the beauty of the distance and the sun tints its borders with gold.

At last, a truly representative Louisiana State Dental Society has been established on a sure foundation and the character, solidity and beauty of the superstructure is entirely committed to your hands. Let no man be recreant to the expectations reposed in him. Let him be steadfast and unflinching in purpose, concentrating every power and resource to the general well being of all around him, thereby elevating himself and through him the profession to which he belongs to the highest rank of public opinion. "There is no difficulty to him who wills." Do not permit yourselves to be turned aside or cast down. Remember that

> "The wise and active conquer difficulties
> By daring to attempt them; sloth and folly
> Shiver and sink at sight of toil and hazard,
> And make the impossibility they fear."

By deep reflection and constant study, practice, and observation the combined endeavors of each and every one of us, with mind and heart enlisted in the cause, cannot fail to rear an edifice which every Louisianian can regard with pardonable pride—symmetrical in all its parts, "a thing of beauty and a joy forever."

The progress and present status of dentistry is mainly due to the efforts of organized bodies. Association has the effect of developing thought; lessening rancour and jealousy; awakening interest in one another; forming friendships and so uniting the profession, and through these combined influences, advancing it in its great and humane work. Fifty years ago "everybody for himself and his satanic majesty take the hindmost" was the motto of the day. Details of practice were regarded as trade-secrets to be jealously guarded and upon no account to be made public. There was no union, or inter-communication of thought, no interchange of ideas. The 18th day of August, 1840, was the natal day of dental societies, when some few prominent men of our profession assembled in New York City and organized the American Society of Dental Surgeons. Then was first seen the uncertain glimmering of that dawn of scientific progress which now encompasses us in the effulgence of meridian splendor. Then was first planted the good seed which now yields so bountiful a harvest and which will continue to blossom forth and fructify until the end of recorded time. What glorious memories, do not the names of Hayden, Bell, Harris, Taylor, Hunter, Townsend, Hullihen and many others recall? These were the intellectual giants whose untiring labors and self-denying zeal first gave to dentistry "a local habitation and a name" and erected our profession upon that proud eminence which it occupies to-day, yielding precedence to none of her sister sciencies. As long as our art will contribute to the aleviation of suffering, to the prevention of pain and to the comeliness and beauty of the human race, so long will they deserve the lively gratitude of posterity. They have long since joined the "silent majority," but their names still live, for they are inscribed upon imperishable tablets in the temple of fame.

To prosper the good work so auspiciously begun, we are convened here to-night. I hope that the love of the profession of your choice and your determination to carry out the purposes of our being as a society, as evidenced by your energy and zeal, will induce every dentist in Louisiana, eligible to its honors, to join this body and participate in its transactions. I do not think a society of educated men must necessarily be a synod of savants, an aristocracy of talent, a first estate in the realm of science; but that it bases itself really upon a conceded republican equality of rights and duties and ought to be democratically representative. Let the profession as it is be fully recognized and let the common interest take care of the common weal. The profession abstractly has a character which is no longer endangered by the unworthiness of pretenders. Admission to membership on open and even conditions is no endorsement of the standing of individuals and works no responsibility of the whole for its parts. Therefore it is of the utmost importance that *all* should rally around the standard and march in solid phalanx to the accomplishment of the great and desirable purposes which lie before us; and among the many duties which present themselves at the present time to us, as a society, none is more urgent than the necessity of securing effective legislation against the inroads of professional incompetents at home and unprincipled adventurers from abroad.

The circular letter drafted at the instance of this body and mailed to every practicing dentist in the State, eloquently sets forth what is expected of the profession towards securing the amendment to the existing law regulating the practice of dentistry (a copy of which accompanied the circular letter), in order that, that law might be wide-reaching and successful in its operation. You have all been made acquainted with what has been done and what is proposed to be done in this regard. The time for action is near at hand. There is no measure that will contribute more to the dignity and good name of the profession. Our own best interests and the interests of the community and common morality demand our undivided exertions.

Let no one then prove unmindful of these demands, but by a determined preseverance in the use of all the means within our reach prepare ourselves to meet all the claims that our profession and the community may have upon us. This being done, success is sure.

Whilst dentistry continues to advance with giant strides, and the benefit it confers on suffering humanity are almost universally acknowledged, it is impossible to conceive the obtuseness and indifference displayed by our public bodies as to its value.

In this city of 250,000 inhabitants, although renowned for the completeness and efficiency of its charitable institutions, there is not to be found a single dental infirmary. In our asylums, establishments where children are sent who are without friends in this world, the services of a doctor of medicine are considered indispensable, whilst the services of the dental surgeon are ignored. Yet how much of human misery and suffering might be obviated and how many useful teeth might be preserved for future use, to the certain increase of health and strength, by the timely aid of the dental surgeon, in detecting and remedying the early predisposing causes of caries and by his careful supervision, especially during the troublesome period of secondary dental eruption, it is impossible to over-estimate. Ought not all public institutions where large numbers of men, women, and children are collected together, to have regularly appointed dental surgeons? The sanitation of our public buildings are superintended and the so-called preventable maladies are duly signaled off; but the rich harvest reaped of dental caries is merely accepted as a painful but necessary phenomenon of life. We maintain it is as much the business of the surgeon-dentist to prevent dental caries as to cure it. This view is adopted by the profession at large and yet the purblind policy, which but too often reigns in boards of directory, fails to see that if they take steps to ward off one group of diseases, it is clearly their duty to guard against the ravages of another. But how? Surely it is not asking a very wide departure from routine, when we desire the appointment of a dental surgeon to every institution, the number of whose inmates render such a course necessary. To commit some two to four hundred children to the inspection of a skilled specialist, say once a month, is not much; yet the extent of benefits accruing to the little patients either in curing or preventing their ills, would be almost incalculable!

It is the province of this Society to shape public opinion in the right direction and influence improvements wherever they shall seem needed. The agitation of this question involves reforms which demand your earnest encouragement. No great social revolution was ever effected without opposition; but the change is near at hand and we may look forward hopefully to the time when mankind will, in this and other regards, recognize the necessity for the dentist and fully appreciate the value of his services.

Notwithstanding our dental colleges are daily sending forth many worthy and thoroughly competent men, the deplorable fact stares us in the face that a great number of young men enter the profession not properly qualified, and in the majority of cases the dentists themselves are to blame. To illustrate: a boy is hired to attend the door bell; in his leisure time he assists in the laboratory; eventually he learns to pull teeth; self-conceit steps in and with swelling pride he concludes he knows more than his preceptor; or the case may be, he is thrown in contact with some ignoramus, practicing dentistry, and he feels his superiority. In either case he hangs out his shingle, a full-fledged dental-surgeon, though, perhaps, never having seen the inside of a dental or medical college, or even read a single treatise pertaining to the practice of dentistry. This is the class which generally produces the advertising dentist, though it must be confessed, with sorrow and shame, that there are some who have drank at the fount of knowledge, so lost to every honorable impulse, so debased in principles as to disgrace their Alma Mater and prostitue their profession for lucre's sake. Advertising Dentists! "By their fruits shall ye known them." An advertising dentist is not a modest man, for he does not "hide his light under a bushel;" he is not a contented man, for he publishes to the world that a generous public has not awarded him his due share of employment; he is not an upright man, for he disregards the edicts of his profession in not obeying its code of ethics; he is an unprincipled man, for he tries to raise his own status by belittling the standing of his fellow practitioners; he is an arrogant man, for he "knows it all." I pity the man who in his arrogance places himself above being taught! he is a conceited man, for he judges others by his own standard; he is a selfish man, for his whole soul is wrapped up in self and to him the almighty dollar attracts every spark of honorable merit; and lastly, he is a dishonest man, for he promises results which he knows can not be attained, and thus procures money under false pretenses.

Indeed the infinite variety of dentists which the various methods of entering the profession has produced, representing every degree of qualification or disqualification for the performance of their important duties towards a confiding public, strongly suggests the exclamation of Macbeth in reply to the assurance of the creatures whom he had engaged "to do a deed of dreadful note" that "we are men, my liege."

> "Ay, in the catalogue ye go for men;
> As hounds, greyhounds, mongrels, spaniels, curs,
> Choughs, water rugs and demi-wolves are 'cleped
> All by the name of dogs; the valued file
> Distinguishes the swift, the slow, the subtle,
> The house-keeper, the hunter, everyone
> According to the gift which bounteous nature
> Hath in him closed; whereby he doth receive
> Particular addition from the bill,
> That writes them all alike; and so of men."

And so of dentists: and this condition of affairs will continue to exist until the means of acquiring the scientific knowledge and manipulative instructions, necessary to properly fulfill the manifold requirements of the art, shall be placed within the convenient reach of every aspirant, desiring to become a worthy member of the profession and until (which must follow as a natural consequence) public opinion shall be enlightened to such an extent that the possession of a diploma from some reputable college will be exacted, as an evidence of competency, before entrusting the teeth to the skill and judgment of any claimant to the public's confidence and patronage. Unfortunately, in many instances, " poverty and not the will consents," and many a man has embarked in active practice, without first procuring a degree; simply because, remote from the centres of education in this particular branch, he had neither the time nor the means to satisfy his laudable ambition.

In the whole United States there are but twenty-one dental colleges, of which eleven are Dental Departments connected with State Universities, and out of the entire number there is not a single one located south of Nashville, Tennessee.

Here at the gateway of the largest, most productive and delightful valleys of the world, spreading itself from the great lakes of the North to the Gulf of Mexico on the South; bordered on the East by the Alleghanies and extending westward to the snow crowned heights of the Rocky Mountains—a garden spot which can sustain and which is filling up with countless millions of freemen, the majority of whom will demand proficient dental services, sits New Orleans, the metropolis of the South, enthroned in the memory of her ancient prestige and splendor, and in the anticipation of greater eminence and glory in the near future; with a population of 250,000 souls, her railways, thousands of miles in extent, radiating in every direction, and the commerce of the Spanish main and of the golden land of the Montezumas ready to fall into her lap; yet, with all these advantages and attractions, it is a remarkable fact that there is not a dental school within a radius of 500 miles of her portals. Reflect on it! But the spirit of progress is abroad. This very building in which we are now convened, originally erected as an institute for the preferment of science and arts, but laterly sadly fallen from grace; first, as a temple dedicated to the votaries of pleasure, then as an arena wherein was enacted many of those disgraceful political struggles which marked with blood Louisiana's reconstruction period, has now been restored to its original use through the beneficence of that noble, generous and good man, Paul Tulane, and which is destined through the wise and judicious management of the distinguished gentleman who has just preceded me to become the mighty University of the South—in excellence second to none on this broad continent. Though at present not complete in all the departments of science, I feel certain that a dental department must and will, in due time, be added to the branches of learning already taught in this admirable institution.

I have rapidly and superficially coursed over a very wide field, yet there are still many subjects which I, in a spirit of forbearance, have left untouched, fearing that I have already sufficiently taxed your patience; but if a single seed out of the many thrown broadcast shall germinate and bear fruit, I shall be amply rewarded in the reflection that I have at least contributed an humble offering upon the altar of our science.

I will conclude by hoping that the utmost good will and consideration for each other will characterize this meeting. Throw aside every feeling but love—love for each other and for that truth upon which the temple of our science must be erected. Labor earnestly.

> "The clouds may drop down titles and estates
> Wealth may seek us—but knowledge must be sought."

Determine to excel and even if the summit of your ambitious views are not attained you will at least place the Louisiana State Dental Society among the excellent of earth and the recognition of its worth will claim the gratitude of posterity.

